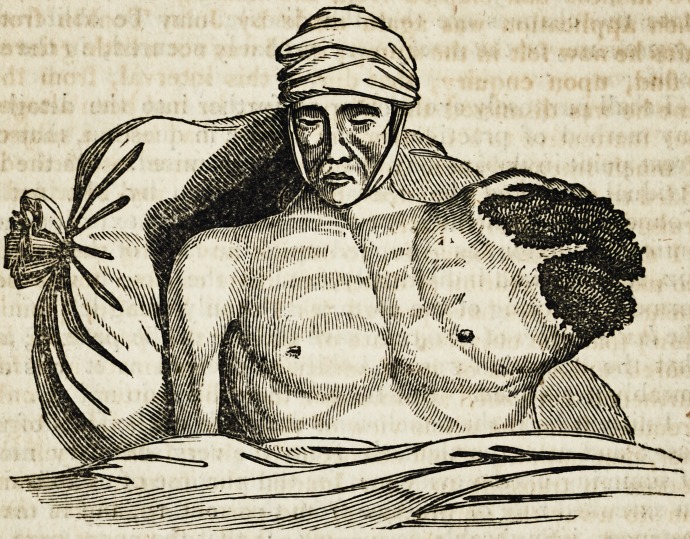# History of a Carcinomatous Affection

**Published:** 1819-05

**Authors:** J. P. Baldy

**Affiliations:** Member of the Royal College of Surgeons; and one of the Surgeons to the Dock and Storehouse Public Dispensary, Plymouth.


*For the London Medical and Physical Journal.*

*History of a Carcinomatous Affection;* by J. P. BALDY, Esq. Member of the Royal College of Surgeons; and one of the Surgeons to the Dock and Storehouse Public Dispensary, Plymouth.

IT has been said by some writer (no matter whom) that niches in the temple of Fame have no constant occupation; the contingencies of human events are in themselves so fluctuating and changeable. But, whether this be true or not in the common circumstances of life, certain it is in relation to what may be justly called, for the time, successful cases in the department of medicine or surgery; the celebrity obtained by them is a tenure, against which, of all others, the *quo warrantos* are most sure to be called forth.

It is a duty which I owe myself, as well as the whole body of diplomatic practitioners in the medical department, amongst whom your Journal is so extensively circulated, that I should recal their attention to a case of peculiar interest, which occupied the first pages of your work for November last, 1818. I refer to the history of JOHN TONKIN, on whom an operation was successfully performed for a carcinomatous* tumor affecting the os humeri. The

* There was an omission on our part in the appellation given to this case on a former occasion: it should have been—" a carcinomatous cartilaginous tumor," it having been so termed by the author.—EDIT.

plan of operation there observed, and the method subsequently pursued until the period of cure, when the patient was dismissed from the Dispensary in apparent good health, have been already stated in that report; it were needless to go over that ground again.

It appears from the records, that somewhat more than two months had elapsed from the healing of the wound, when application was again made by John Tonkin from pains he now felt in the stump; and I was not a little grieved to find, upon enquiry, that during this interval, from the time he was dismissed the Dispensary, he had been altogether inattentive to his manner of living, had been a subject of much intemperance, and more than once intoxicated: added to these circumstances, he had fallen, and received a bruise in the affected part.

I do not mean, however, to ascribe any thing more than the mere aggravation of disease to these causes; for subsequent discoveries have proved (as I shall hereafter plainly show) that the universal state of malady this poor man laboured under was of itself sufficient to terminate any life burthened with such accumulated morbific matter. I only premise, from the whole view of this case when taken together, that nothing which has occurred since I did myself the honour to transmit to your Journal the former statement can do away the impression of the correct treatment then observed, when both the operation and subsequent cure of the wound ended with so much success.

Recalling as much of your attention to the case of this man as may be needful to form an accurate judgment of what was, and what was not, connected with the original disease, I have to observe that, on the 13th day of August last, (a period of more than two months from the time of his cure,) I was called upon to visit John Tonkin, who complained of a considerable degree of pain in the stump, attended with an increased temperature of the parts.

I considered it a subject of some moment to ascertain whether any symptoms unfavourable had taken place since he left the Dispensary, and found a most satisfactory answer to my questions, that nothing had arisen unfavourable during the whole interval. He expressed himself as one who had been perfectly free from pain, and, in his own view, as good in health as in any former period of his life. I discovered, however, what I before observed of his irregularity of living, and that he had been occasionally overtaken (as he expressed it) in liquor; during one of which seasons he fell, and knocked his stump.

I judged it right to order an evaporating lotion to be constantly applied, accompanied with a brisk cathartic; hoping thereby to lessen the tension and suppress all inflammation.

The following day, I found the purgative had fully accomplished its purpose, although the pain of the part was not lessened, and the heat rather increased than diminished.

Matters continued much in the same state until the 20th, when, on visiting my patient, I found the heat not so great, but the integuments below the acromion, as well as those covering the pectoral muscle, began to swell; but, as all these symptoms, however alarming, were unaccompanied with fever, there appeared no indication of any thing immediately to be done; I therefore recommended the constant application of the lotion, and waited the result.

The swelling continued to increase in size, but with little or no pain, until the 3d of October; when, early in the morning of that day, I was sent for in great haste on account of hæmorrhage. On examination, I found that an ulceration of the integuments had taken place, through which the blood was issuing in a stream, about the size of a crow's quill. Happily the bleeding was stopped by thick compresses of lint and bandage.

The disease, in the nature and extent of it, which had hitherto remained unknown, now developed itself, and in such characters as left no room to doubt of its being that formidable and incurable malady which Mr. Hey very properly has denominated Fungus Hæmatodes, although misnamed cartilaginous.

Throughout the whole of this day there was a considerable degree of fever; pulse 110; tongue coated; and a great dryness of the skin. I was again sent for in the evening, hæmorrhage having again recurred: the former plan was again resorted to with success, and the parts kept wet with a solution of alum.

The swelling under the acromion now assumed a very formidable appearance, and threatened to burst through the integuments every hour.

4th.—Fever still very high; pulse 141; tongue furred; great anxiety; but no pain in the tumor.

5th.—Had no sleep during the night; fever still the same; pulse 140. On removing the compresses, bleeding again ensued in a very large stream: it was again compressed as before, but the tumor had considerably increased.

In the evening I held a consultation of my colleagues, (for the first time since the operation,) the result of which was, that nothing in a curative way could be adopted; but, in case of hæmorrhage, where the compression did not succeed, styptics should be employed. My patient this evening was very low; fever high; pulse 140. An opiate was ordered.

6th.—Had a very restless night; pulse 140; examined the tumor, found the ulcerative process of the integuments going on very rapidly; no hæmorrhage; dressed it as before.

7th.—Fever little abated; pulse 126. The dressings were allowed to remain as the day before.

8th.—Fever less; pulse 110; examined the tumor, the ulceration of the integuments still continuing.

After this, his general health began to improve; but the tumor continued to increase in size, with a great enlargement of the parts covering the pectoral muscle, till the 26th of October, when a large portion of integuments gave way, and left *in situ* a large fungus, from which constantly oozed a bloody serous discharge.

I pause here to observe, but with due deference to Mr. Hey's opinion in the case of Mrs. Dean, that the fungus, as it increases in size, does not render the integuments uniformly thin, as in abscess,—that in this instance it differed. The skin became of a dark-red colour, and uniformly thin, as in the case of abscess.

From about this time my patient's health began again to decline, and that rapidly. He had, indeed, to encounter many extraneous matters beside disease, all highly disadvantageous. Poverty is, for the most part, attended with a train of evils. The very situation in which he lived, in an underground room beneath the street, rendered the air damp and impure; and the fœtor from the wound formed a constant effluvia to add to the offensive atmosphere.

The fungus from this time continued to increase in size very rapidly, and resembled in appearance a putrid placenta, from which a serous bloody discharge continually oozed, sufficient in quantity to soak through the bed that lay under him.

November 12th.—About this period, the integuments at the lower part of the stump gave way, similar to the one above; and so rapid was this in its growth, that, by the 15th, a portion sloughed weighing three ounces. From the increased quantity of discharge, I was now obliged to dress him thrice a day, and at each dressing always a quantity of blood escaped.

20th; the last day of his life.—At ten o'clock I again dressed him, when the fungus appeared as if it would slough every moment. Vomiting now came on, so that he could not be supported up in his bed without being subjected to it.

At eleven o'clock I was obliged to dress him again, on account of hæmorrhage, as if from a sponge, to a very great extent. At half-past four o'clock I again dressed him, for the last time, when a portion of the fungus came away, weighing fourteen ounces and three quarters, unaccompanied with any hæmorrhage. He at this time had dyspnœa, and appeared sinking fast, having singultus and vomiting. During the evening, however, he spoke very cheerfully to his friends, but was getting lower and lower, when, at a quarter after eight, he expired.

So interesting a case furnished a most desirable subject for dissection, in which we were gratified upon examination.

*The Appearances on Dissection.*—On examination of the chest, we found the pleura considerably thickened, and the lungs slightly inflamed, but unattended with strong adhesions. The lungs were also studded with tubercles, of precisely the same consistence as the tumor. Between the fourth and sixth ribs, a whitish substance appeared, which, on cutting into, proved to be of the same nature as the disease. The abdominal viscera exhibited a healthy appearance, excepting the liver, on which was a tubercle similar to that on the lungs. The glenoid cavity was very much diseased. The tumor itself was of a soft medullary nature, not unlike the brain after being kept a week exposed to the air.

Having with great faithfulness stated the whole of this peculiarly interesting case, I shall now, with great cheerfulness, leave the decision to the judgment of that body of practitioners before whom this Medical Journal shall come.

There are several observations not a little singular with which this case was attended, which, no doubt, in common with myself, will strike the judicious and discerning reader. Desperate as the disease was in its early stages, it is very strange that so long a period of two whole months of apparent health and strength should have taken place, and actually to have suspended, during that interval, the progress of disease to the sure and fatal end. And it is equally wonderful, in the discovery made by dissection, that, during the whole of this formidable malady, notwithstanding the great pressure of the tumor, and the tubercles in the lungs, he had neither pains in the chest nor any difficulty of respiration, till within four hours of his death. And it is not the smallest circumstance worth noticing, that the ribs were not the least diseased, although the tumor lay completely on them.

In relation to the patient himself, his mind appeared to have been fortified both for the pains in the operation and the several preludes of agony he passed to the grave; and, had he lived longer, young as he was, with lungs so diseased, a mutilated body, a disposition to disease in the viscera, and no prospect of maintenance from labour, whatever way he looked, the view would have been dark and gloomy. Cicero hath finely expressed it—" Nihil ei beatum cui semper aliquis terror impendeat."

*Plymouth Dock*; *March* 4, 1819.

P. S. I think it right to notice that an error was inadvertently made in my report of the consultation held on the above case, on the 15th April. Your readers will observe, it is there stated to have been the unanimous opinion that the tumor contained a fluid; whereas, I now learn from my colleagues who were present, that the existence of a fluid was only deemed *probable* from the great elasticity of the tumor, and that the prevailing opinion was in favour of an opening being made, which would relieve the patient from the pain of distension, in case the tumor did contain a fluid, and would enable us, perhaps, to judge more correctly of the nature of it.

## Figures and Tables

**Figure f1:**